# Revisiting pancreatic islet isolation in murine models: A practical and effective technical protocol

**DOI:** 10.14814/phy2.16040

**Published:** 2024-05-09

**Authors:** Aline Fernandes‐da‐Silva, Rosiane Aparecida Miranda, Patricia Cristina Lisboa, Vanessa Souza‐Mello

**Affiliations:** ^1^ Laboratory of Morphometry, Metabolism, and Cardiovascular Diseases, Biomedical Center, Institute of Biology Roberto Alcantara Gomes State University of Rio de Janeiro Rio de Janeiro Brazil; ^2^ Laboratory of Endocrine Physiology, Department of Physiological Sciences, Institute of Biology Roberto Alcantara Gomes State University of Rio de Janeiro Rio de Janeiro Brazil

**Keywords:** endocrine pancreas, isolated islets, methodology, pancreatic islets

## Abstract

The endocrine pancreas is composed of clusters of cell groups called pancreatic islets. These cells are responsible for the synthesis and secretion of hormones crucial for glycemic homeostasis, such as insulin and glucagon. Therefore, these cells were the targets of many studies. One method to study and/or understand endocrine pancreatic physiology is the isolation of these islets and stimulation of hormone production using different concentrations of glucose, agonists, and/or antagonists of specific secretagogues and mimicking the stimulation of hormonal synthesis and secretion. Many researchers studied pancreatic physiology in murine models due to their ease of maintenance and rapid development. However, the isolation of pancreatic islets involves meticulous processes that may vary between rodent species. The present study describes a simple and effective technical protocol for isolating intact islets from mice and rats for use as a practical guide for researchers. The method involves digestion of the acinar parenchyma by intraductal collagenase. Isolated islets are suitable for in vitro endocrine secretion analyses, microscopy techniques, and biochemical analyses.

## 1 INTRODUCTION

The endocrine portion of the pancreas is small, and it is composed of the islets of Langerhans, also known as pancreatic islets (Leung, 2010; Navarro, 2014). The islets are spread throughout the acinar parenchyma and primarily include five types of endocrine cells that produce and secrete the hormones responsible for important biological functions. β cells make up 60–80% of the islet population and are the most important cells for understanding insulin secretion. The α cells secrete glucagon, and δ cells secrete somatostatin. The ε cells secrete ghrelin, and PP cells secrete pancreatic polypeptide. These different cells are architecturally arranged to allow functional communication between islets, which may be orchestrated via autocrine, paracrine, and endocrine signals (de Cassia Gonçalves et al., 2023).

Viable pancreatic islets are important for research involving cellular, molecular, and functional aspects of the endocrine pancreas, and correct islet isolation is a crucial prerequisite. Proper isolation depends on many factors, such as the strain, weight/age of the animal used, sex, sources of bovine serum albumin, collagenase composition, culture conditions, and different methods, that determine the effectiveness of isolation and improve the yield (de Haan et al., 2004; Sabek et al., 2008).

Although there are some techniques for isolating pancreatic islets, they are different methodologies with particularities. Most require a greater number of reagents, equipment, and cannulation methods that are difficult to apply in a small animal model such as mice or weaning rats. Furthermore, there are no methodological articles demonstrating the differences between isolation techniques between mice and rats. Therefore, the current study presents our standardized method for the isolation of pancreatic islets in mice and rats in detail to reduce the variation and increase the effectiveness of the technique without compromising the structural and functional integrity of the cells.

### 1.1 The pancreas

The pancreas is a mixed gland, and the endocrine portion is helpful for ex vivo experiments to assess insulin secretion and perform Western blotting (WB) or RT‐qPCR (Ramírez‐Domínguez, 2016). The mouse pancreas has a compact splenic portion and a duodenal portion that is diffused within the mesentery. The endocrine pancreas comprises only 2% of the total pancreatic mass (Dolenšek et al., 2015). One challenge of the islet isolation protocol is the digestion of the exocrine portion of the pancreas while maintaining viable and functional islets for in vitro or in vivo experiments (Villarreal et al., 2019). Correct identification of the common bile duct and the ampulla of Vater are critical steps for successful islet isolation once most islets are found in the splenic region. Therefore, clamping the common bile duct near the ampulla of Vater allows a complete perfusion of the pancreas (O'Dowd, 2009).

**FIGURE 1:**
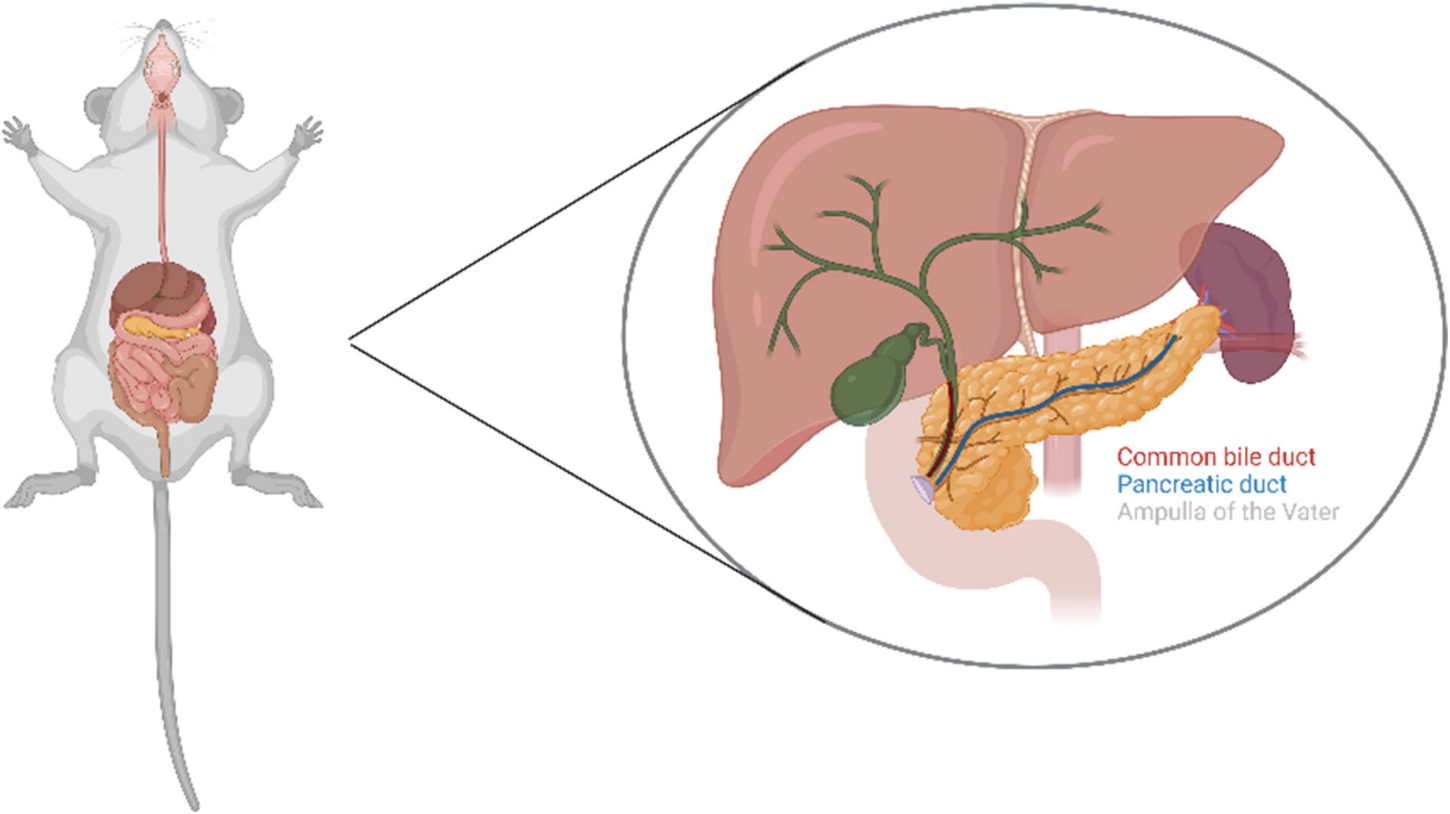
Schematic illustration of the organs in the mice's abdominal cavity with signs of the common bile duct, pancreatic duct, and the region of the ampulla of the Vater, crucial structures whose identification guarantees the technique's success. Of note, the rat anatomy resembles a mouse, except for the absence of the gallbladder (Kruepunga et al., 2019). Made with Biorender (www.biorender.com).

The liver secretes bile, and the gallbladder stores it until the presence of fat in the duodenum stimulates its release. The common bile duct, which is responsible for carrying bile, results from the union of the common hepatic duct and cystic duct. It meets the pancreatic duct, which carries the digestive juices stemming from the exocrine pancreas, in the ampulla of Vater. The digestive juices and bile reach the duodenum through the major duodenal papilla to accomplish food breakdown after it leaves the stomach (Hundt et al., 2024). Correct clamping of the common bile duct to perform islet isolation relies on exposure of the posterior liver region to identify and clamp the common bile duct near the duodenum. The cannulation should target the Y formed by the cystic duct and the left hepatic duct for injection of collagenase type V and insufflation of the whole pancreas (from the duodenum to the splenic region), as shown in Figure 1.

After the digestion and collection steps, which are described in detail in the following sections, the isolated islets are ready for insulin secretion tests or in vitro experiments and molecular analyses (stored at −80°C). Notably, approximately 200 islets per animal are sufficient to perform RT‐qPCR, and Western blotting requires approximately 300 islets per animal. The current protocol provides approximately 250–400 functional and viable islets from mice and 500–700 from rats.

### 1.2 Insulin secretion

Pancreatic islets have multi‐hormonal secretion, but insulin secretion is a frequent target of studies due to its close relationship with the diagnosis of insulin resistance and the onset of type 2 diabetes mellitus (Prentki & Nolan, 2006). Pancreatic beta cells are polarized with a well‐developed secretory ultrastructure that includes a rough endoplasmic reticulum and Golgi apparatus in the pole opposite the nucleus (Souza‐Mello et al., 2011). Insulin has an autocrine/paracrine role in pancreatic beta cells because insulin binds to its receptors in the beta cell membrane, and the consequent postreceptor signaling to GLUT‐2 translocation allows glucose to enter the cell. Oxidative phosphorylation increases intracellular ATP levels and triggers ATP‐sensitive K+ channel closure, membrane depolarization, and calcium influx, which is the stimulus for insulin granule release. Any perturbations in insulin secretion, including insulin signaling within the beta cell or dysfunctional organelles involved with insulin synthesis and posttranslational modifications, compromise glucose‐stimulated insulin secretion (GSIS) (Komatsu et al., 2013; Lu & Li, 2018; Prentki et al., 2013). The insulin secretion test addresses these events.

### 1.3 Different techniques

There are some techniques for digesting the exocrine pancreas and obtaining islets, and the following main methods are used:

1. Removal of the pancreas then cutting it with scissors to expose its surface, followed by the use of collagenase for enzymatic digestion in association with manual agitation for mechanical digestion (Prentki et al., 2013; Sutton et al., 1986).
2. Injection of collagenase into the common bile duct, inflation of the pancreas in situ, and subsequent excision to perform the digestion process at 37°C (MacLennan et al., 1953; Sutton et al., 1986).
3. Injection of collagenase into the ampulla of Vater to reach the common bile duct. In this case, the common bile duct is clamped as close to the liver as possible, which perfuses the pancreas in the opposite direction of technique 2 (Villarreal et al., 2019).

Although these techniques are primarily used, there are numerous other modifications due to the unique characteristics of each animal model, which must be considered when using a specific protocol.

## 2 MATERIALS AND METHODS

### 2.1 Our technical protocol

We used two experimental rodent models: male C57BL/6 mice and male Wistar rats (*Rattus norvegicus*).

To obtain this protocol, we tested different types of solutions, times, and steps used in other methods and standardized these three simple steps for our experiments until islets were obtained: in situ cannulation of the pancreas with collagenase, digestion of the pancreas, and collection of islets.

### 2.2 Required material

**FIGURE 2:**
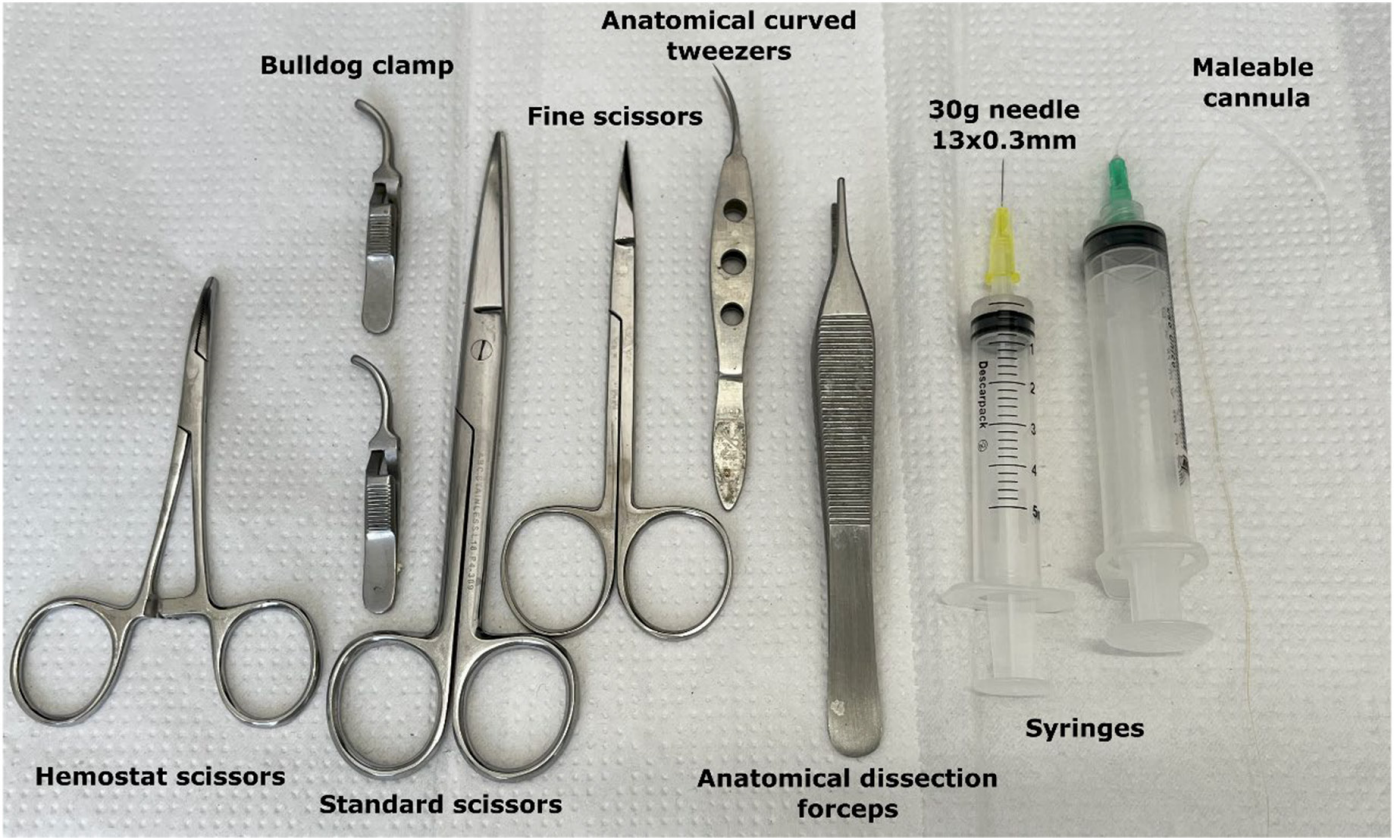
Surgical materials used for pancreatic islet isolation. In rats, the technique requires a malleable cannula connected to a syringe. Differences regarding the materials used for mice encompass a bulldog clamp and the 30G needle coupled with a small syringe.

The following materials listed below and illustrated in Figure 2 are suitable for mice and rats, with differences noted where necessary:

- Styrofoam box with ice
- Beaker for HBSS and collagenase solution
- Gauze with 70% alcohol
- 1–2 bulldog clamp or hemostatic forceps
- 1 pair of standard scissors
- 1 pair of fine scissors for internal use
- 1 pair of anatomical dissection forceps
- 1 pair of anatomical curved tip tweezers
- 30G needle (13 × 0.3 mm) for mice; malleable silicone or plastic cannula attached to a needle hub for rats (improvised material by removal of the needle and attachment to the cannula)
- 5‐mL syringe for mice; 10‐mL syringe for rats (1 per animal)
- Conical tube, 15 mL (1 per animal)
- Petri dish with a black background for islet quantification
- Water bath filled with distilled water at 37°C
- Carbogen, which is a mixture of carbon dioxide (CO_2_) and oxygen gas (O_2_): v/v; 95% O2 C5% CO_2_ for pancreas digestion and islet incubation
- Binocular stereomicroscope
- Pipettes and tips, 200 μL and 1000 μL
- Microtubes, 1.5 mL, for storage of the supernatant content

### 2.3 Solutions

#### 2.3.1 Hank's balanced salt solution (HBSS)

We standardized 50 mL of the solution for each mouse and 200 mL for each rat, considering the content for collagenase dilution, the washing after digestion, and the volume needed to collect the isolated islets.

#### 2.3.2 For 1 L of HBSS solution

1. NaCl (sodium chloride)—8 g
2. KCl (potassium chloride)—0.4 g
3. MgSO4 7 H_2_O (magnesium sulfate)—0.2 g
4. Na2HPO4 (Dibasic sodium phosphate)—0.048 g
5. KH2PO4 (Monobasic sodium phosphate)—0.06 g
6. CaCl_2_ 2H_2_O (calcium chloride)—0.1854 g
7. NaHCO_3_ (sodium bicarbonate)—0.35 g

#### 2.3.3 Method of preparation

Half of the final volume of distilled water was added to a beaker and placed on a stirring plate (Ex: to make 1 l, 500 mL of water was added). The reagents were added in numerical order, and each reagent was diluted before the next reagent was added. The volume was transferred to the test tube and filled to the final volume. Carbogen (v/v; 95% O2 C5% CO_2_) may be infused into the solution for 10 minutes as an optional step. If carbogen is used, NaHCO_3_ (reagent 7) should only be added after this step. The pH was adjusted to 7.4. Hanks' solution can be stored for up to 1 week under refrigeration at 4°C.

On the day of the experiment/islet isolation, glucose, and BSA were added to the solution at the following concentrations:

1. Glucose (0.1%)—0.5 g for 500 mL (or 1 g for 1 L of HBSS).

2. BSA (0.1%)—0.5 g for 500 mL (or 1 g for 1 L of HBSS).

The previously prepared solution was poured into a beaker on a stirring plate. The last reagents were added in order, waiting for each reagent to dilute before the next reagent was added.

Note: Shaking more slowly prevents the formation of bubbles in the solution.

The pH, which should be 7.4, was checked. The pH was adjusted with acidic HCl or a basic lower concentration of NaOH if needed.

This solution was stored on ice throughout the experiment.

#### 2.3.4 Important notes

1. It is important to remember that 50 mL must be calculated for each mouse and 200 mL for each rat when determining the volume of HBSS needed, and from there, use the rule of 3 to determine the amount of each reagent needed. The quantities described above are 1000 mL.
2. The 50 mL or 200 mL per animal was calculated as 30 mL for the bath (rounding off) + 6 mL collagenase +14 mL for cell quantification) for mice and 40 mL for washes between the decantation (rounding off, considering 4 washes) + 10 mL for collagenase +30 mL for quantification) for rats.

### 2.4 Collagenase

Collagenases are proteolytic enzymes that digest collagen, which is an important structural protein in animals (Harper, 1980; MacLennan et al., 1953).

The proper selection and use of collagenase is crucial for digestion because the enzyme degrades the connective tissue around the pancreatic islets (Sawada et al., 2003; Shimoda et al., 2012) and contributes to the success of the technique.

Collagenase was obtained from *Clostridium histolyticum* (Cl. histolyticum) (Jennison, 1945), which is the bacterium used to purify collagenase for the first time (Mandl et al., 1953) and is capable of degrading collagen and collagen‐like proteins.

Clostridium histolyticum has the colG gene, which encodes class I collagenases, and the colH gene, which encodes class II collagenases (Matsushita et al., 1999). The class of collagenase is defined based on substrate specificity and amino acid analysis.

There is great variability between manufacturers and production batches of collagenase because it is a product of bacterial culture. This variation affects the enzymatic activity and the isolation results. Therefore, we standardized the use of type V collagenase (C9263, Sigma Aldrich, St. Louis, USA) and the quantity used. When necessary, we varied the digestion time based on the characteristics of the tissue throughout the process. For example, the digestion time of each pancreas may vary depending on the treatment to which the animal was exposed. Adult rats that were exposed to a low‐protein diet during puberty needed a shorter digestion time, approximately 6 minutes less, compared to their controls because their pancreas was structurally compromised (control diet: 17 min and low‐protein diet: 11 min) (de Oliveira et al., 2013). It is important to consider the treatment, age, and sex of the animals because these factors also affect digestion time. Therefore, we suggest testing each batch for optimal islet viability and function for successful and reproducible islet isolation.

#### 2.4.1 Reagents used for collagenase solution

- N‐2‐hydroxyethylpiperazine‐N‐2‐ethane sulfonic acid (HEPES) 0.6%
- Collagenase type V: 1 mg/mL
- BSA 5%

For each mouse, 6 mL of HBSS (for collagenase dilution) was used. For each rat, 10 mL was used. Example: 2 mice = 12 mL of collagenase solution, therefore, it would be necessary to add:

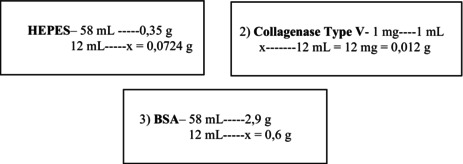

Note: Ideally, HBSS should be prepared on the day of surgery, and the collagenase solution should be diluted in HBSS no more than 2 h before surgery.

### 2.5 Cannulation, digestion, and collection of isolated islets

#### 2.5.1 Cannulation

**FIGURE 3:**
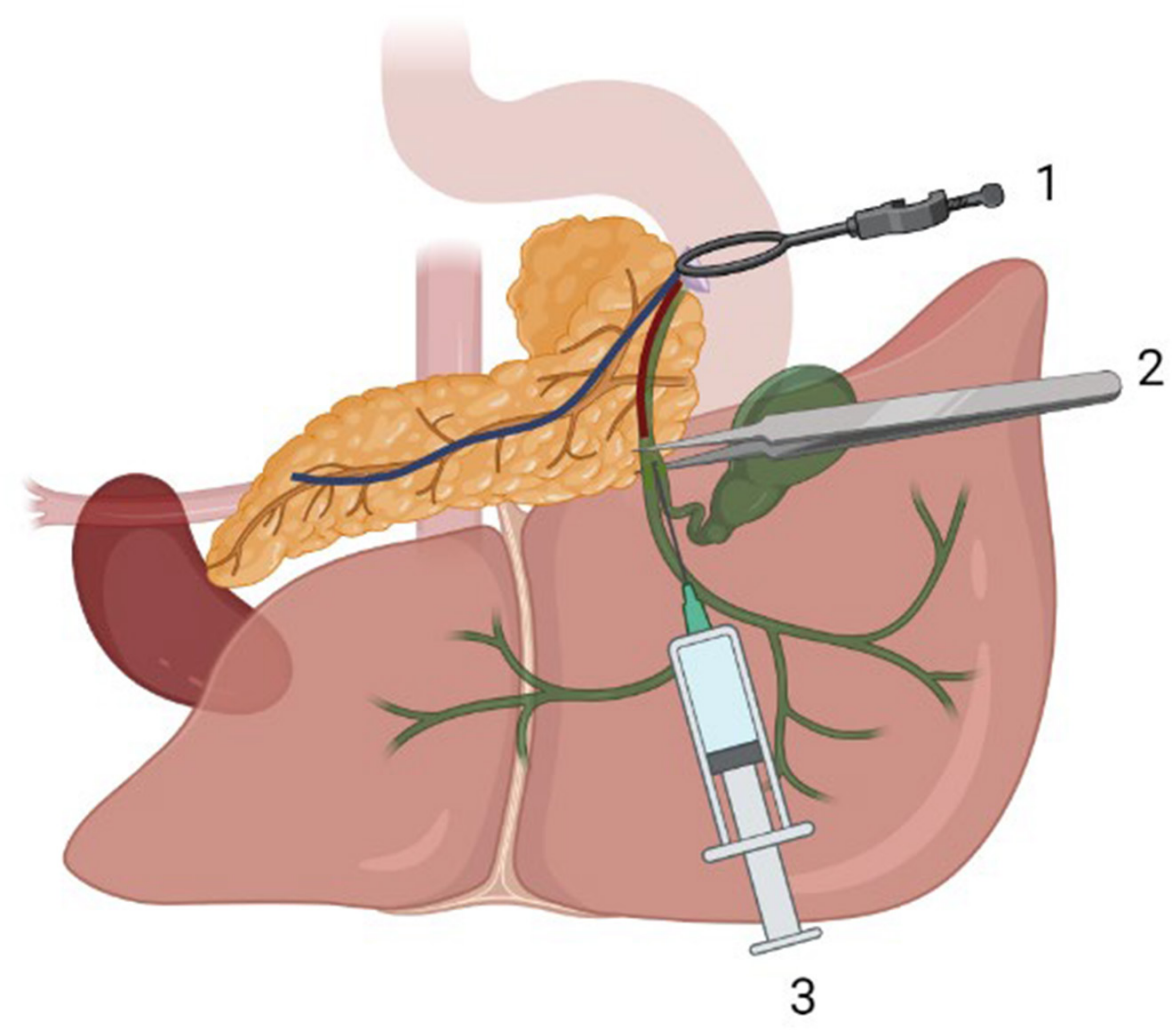
Representative diagram of the location in the small intestine where clamping is performed (Navarro, 2014), identification of the common bile duct with the help of forceps (Leung, 2010), and cannulation with the needle (de Cassia Gonçalves et al., 2023) in mice. It is important to highlight that during the procedure, the experimenter must ensure the stability of the surgical table and his hands to ensure that no vibration could disrupt the cannulation of the duct. Furthermore, the experimenter should avoid multiple injections into the duct region of the mice, as it is a very thin portion and can easily rupture. Thus, the injection can be taken more distally at the duct. Made with Biorender (www.biorender.com).

1. Animals were euthanized using CO_2_ gas or decapitation. Both techniques produce only minor alterations in insulin secretion by islets, unlike the use of anesthesia.
2. The animal was completely exsanguinated via cardiac puncture in the event of fainting due to CO_2_, to avoid excess blood in the pancreas, which compromises the technique. This step is extremely important for the success of the technique.
3. Before the surgical cutting, the animal's entire body was cleaned with 70% alcohol to prevent hair from sticking to the intraperitoneal cavity.
4. When the animal did not respond to the paw pinch test, an incision was made with scissors starting in the lower part of the abdomen (close to the genital region) and extending to the lateral portions of the diaphragm to expose all of the organs in the peritoneal region and the heart, with extra care to avoid damage to the liver.
5. The animal's head was positioned close to the handler's body to align the anatomical position of the duct for cannulation.
6. The spleen was identified because the tail of the pancreas is associated with this tissue. This region was fully inflated. This area is farthest from the needle insertion point. During the procedure, it is necessary to check for perfusion of the tail of the pancreas. The liver lobes were moved to the sides to expose the common bile duct.
7. The common bile duct was identified in the duodenum, and the bulldog clamp was used to block access of the contents leaving the duct to the small intestine region. If the contents return to the liver, clamp or suture the area of the duct before cannulation.
8. A 5‐mL syringe was filled with the collagenase solution for mice, and a 10‐mL syringe was used for rats. It is important to make sure there is no air in the needle.
9. For stability when using curved fine‐tipped forceps, the common bile duct was isolated from below. Furthermore, it is important that the researcher has their elbows and forearms resting on the table. Removing the connective tissue around the duct can also help prevent the needle from slipping during cannulation. Use the dominant hand to inject the collagenase solution into the duct. Additionally, the nondominant hand can assist (with a forceps) in stabilizing the duct during the injection of the collagenase solution.
10. The common bile duct was cannulated with the 30G needle attached to the 5 mL syringe for mice and with the malleable cannula for rats from the junction of the cystic duct of the gallbladder (mice) and the left hepatic duct of the liver (where it forms a “Y”), as illustrated in Figure 3.

**FIGURE 4:**
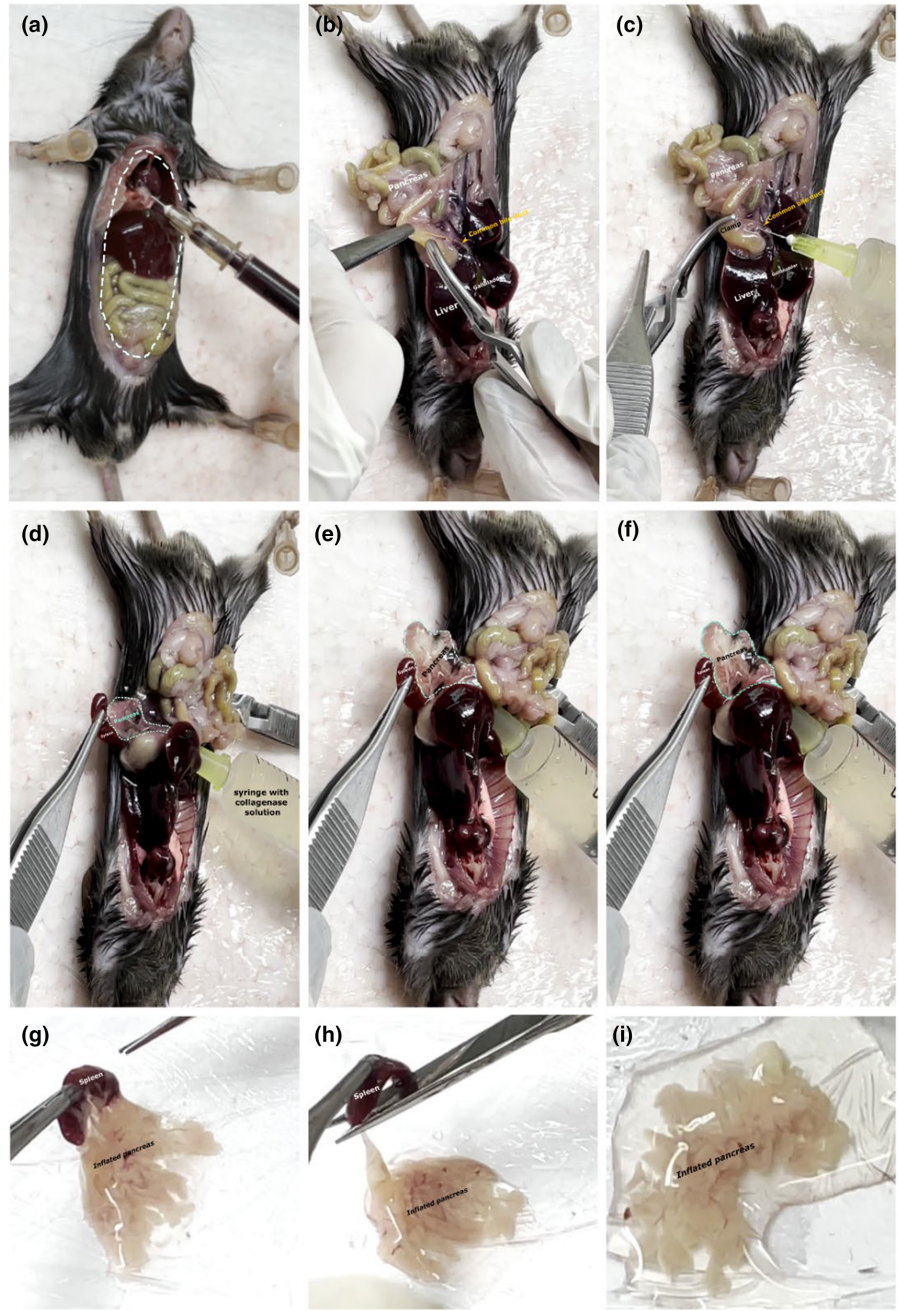
Representation of the steps for cannulating the mouse pancreas with the collagenase solution. Be sure to expose the abdominal cavity to access the pancreas easily (a). When identifying the duct (b), position the needle and ensure there are no bubbles (c) using the spleen as a reference to ensure that the entire pancreas will be inflated (d–f). Afterward, we recommend removing the pancreas with the spleen, which can be discarded later (g–i). For these photos, we used a male mouse approximately 180 days old.

**FIGURE 5:**
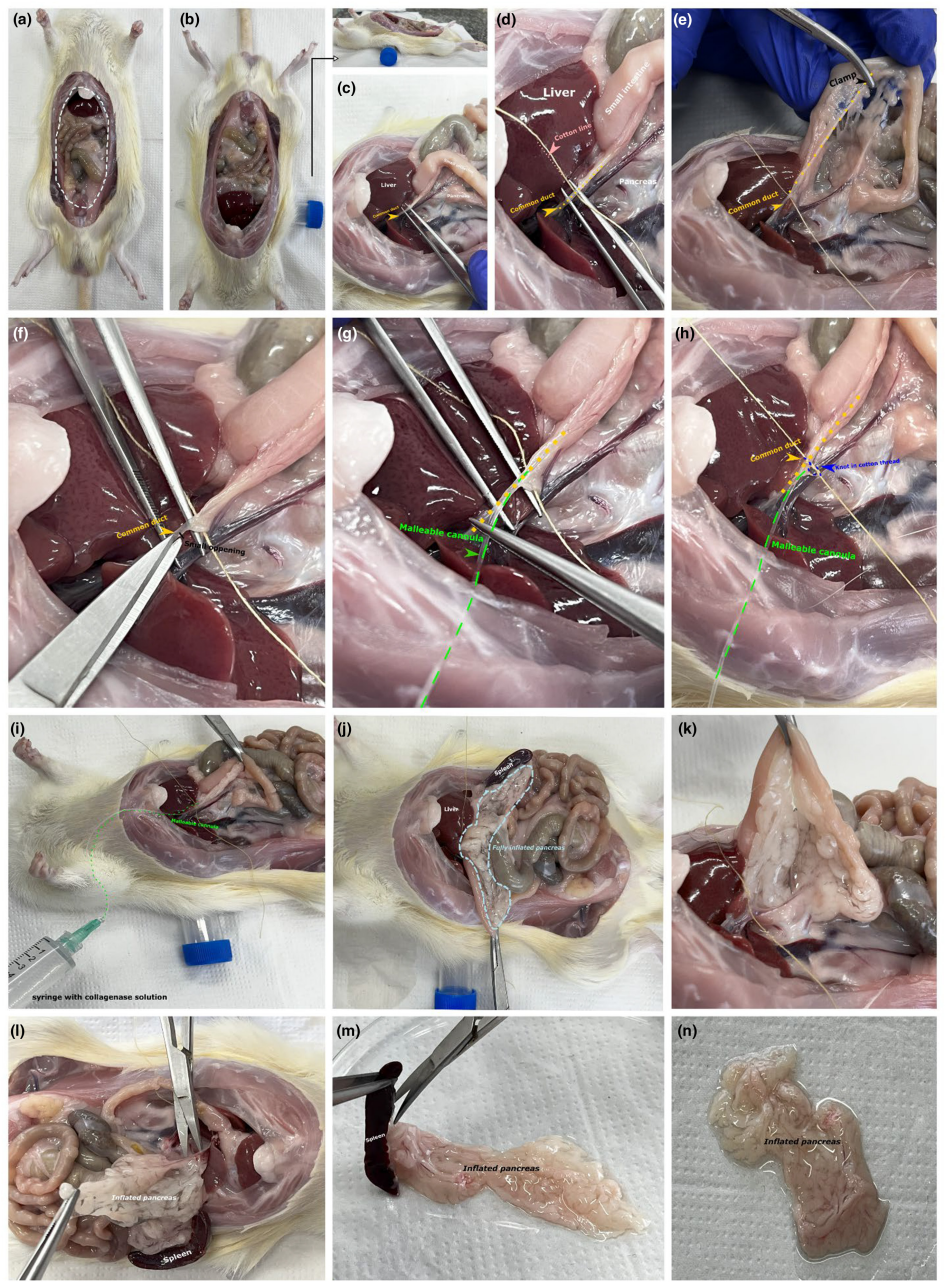
Representation of the steps for cannulation of the rat pancreas with the collagenase solution. Be sure to expose the abdominal cavity to easily access the pancreas (a and b). For better visualization and exposure of the duct, in addition to ensuring a better mobility area for the experimenter, we placed a 15 mL falcon tube on the back of the rat as indicated by the arrow (b). When identifying the duct (c), isolate it and remove excess connective and/or fatty tissue that surrounds the duct, pass a piece of cotton thread under the duct (d). Observe the final portion of the duct that meets the intestine and clamp it to avoid leakage of the collagenase solution (e). With your nondominant hand, stabilize the duct with a forceps, and with your dominant hand make a small incision in the duct (f). It is important to be extremely careful not to tear the duct. Insert only the tip of the malleable cannula (about 5 mm) (g). Wrap the part of the cannulated duct with cotton thread and tie a knot to ensure the stability of the cannula (h). Inject the collagenase solution with a syringe attached to the cannula, avoiding bubbles (i), and use the spleen as a reference to ensure that the entire pancreas is inflated (j and k). Afterward, we recommend removing the pancreas along with the spleen, which can be discarded later (l‐n). For these photos, we used a male rat approximately 120 days old.

As shown in Figures 4, 5, the needle or cannula was oriented parallel to the duct. At this time, it is important to ensure that the connective and fatty tissue surrounding the duct is removed, as this may interfere with cannulation. Then, the solution was injected to completely inflate the head, neck, body, and tail of the pancreas. The dominant hand should be used to inject the collagenase content into the pancreas, and the nondominant hand should be used to move the intestinal loops to the side, pinch, and pull the spleen to expose as much of the pancreas as possible to ensure that the entire organ is being inflated. Additionally, the nondominant hand can assist (with a forceps) in stabilizing the duct during injection of the collagenase solution.

This step allows the collagenase to contact a greater surface area of the organ and perform a better digestion process. It is important to change the needle for each animal cannulation. For mice, the amount of solution injected will vary from 3 to 5 mL, depending on the size of the animal, and for rats, it will vary from 8 to 10 mL.

The pancreas must inflate from head to tail, but the volume of the solution should not cause the pancreas to burst accidentally. At this stage, if the duct ruptures or the pancreas bursts, collagenase is injected directly into the various lobes of the organ in a volume that is sufficient to fill the entire pancreas.

Another possibility is the removal of the pancreas and adding it to a dry beaker. The pancreas may be pricked with scissors, and approximately 2–3 mL of the collagenase solution may be added for mice and rats.

The process of digestion with collagenase using the animal's anatomy guarantees a reduction in mechanical damage to the islets, and studies show that when injected into an intact pancreatic ductal system, collagenase interacts better with the connective tissue surrounding the islets, which increases the effectiveness of the isolation (MacLennan et al., 1953; Mandl et al., 1953).

#### 2.5.2 Digestion

**FIGURE 6:**
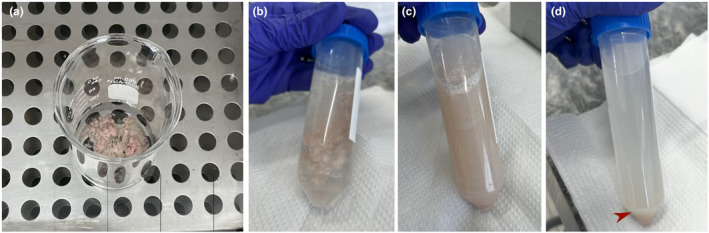
After removing the pancreas and taking it to the beaker, the digestion process will occur by collagenase (a), and HBSS addition interrupts the process (b). After being shaken (c) to dissociate the tissue, it goes through the pellet decantation process (d).

11 Once inflated, the pancreas was removed from the abdominal cavity using blunt forceps to carefully separate it from the intestines and avoid rupture of the intestines to prevent contamination and rupture of the pancreas. The pancreas was carefully removed from the stomach and spleen and placed in a dry beaker. It is important to ensure that the time until total removal of the inflated pancreas is as short as possible to avoid drying of the organ and incorrect digestion time.
  A small amount of collagenase was added to the pancreas in the beaker to moisten the tissue from the outside (1–3 mL).
  Be careful not to remove any fatty tissue by mistake. In general, adipose tissue is whiter in color than pancreatic tissue, and the adipose tissue tends to float after the addition of the collagenase solution.
12 The beaker was placed in a 37° water bath, stirred by hand with gentle circular movements, and the bottom of the beaker remained in contact with the hot water for approximately 8 minutes. As an optional process that may improve the viability of pancreatic islets, this step may be performed under oxygenation (v/v; 95% O_2_C_5_% CO_2_, mixed).
  Collagenase at room temperature digests the exocrine tissue, and stirring contributes mechanically to the process.
  At this stage, the contents will become cloudy and more homogeneous (Figure 6) due to autolysis of the acinar cells. It is important to observe to ensure that the pancreas has lost its structure and has become visibly digested. In some cases (e.g., based on the size of the animal or type of diet), a longer time is needed (approximately 10 min), so observation of the visual characteristic of the homogenate is important.
  In addition to exogenous collagenase, endogenous proteolytic enzymes released during the digestion process by the exocrine pancreas influence digestion. The addition of BSA to the solution helps suppress these enzymes (Mandl et al., 1953; Matsushita et al., 1999) and provides nutrients for the isolated islets.

Any remaining adipose tissue should be removed using tweezers.

13 The digested pancreas was transferred to a falcon tube, and it was filled with 8 mL of HBSS for mice or 40 mL for adult rats. HBSS was used to stop the digestion process.
14 The capped falcon was shaken vigorously by hand for 1 minute or until the pancreas completely lost its shape, and a homogeneous mixture was obtained. This step is very important to cause dissociation of the pancreatic tissue by enzymatic degradation of the extracellular matrix without damaging the integrity and functionality of the islets.
15 This solution was decanted on the bench for approximately 10 min.
16 The excess “dirty” HBSS was transferred to a larger falcon, and only approximately 5 mL remained in the tube. Visually, a white mass settled at the bottom of the tube.
17 The volume was adjusted to 8 mL with HBSS for mice or 40 mL for adult rats and mixed for 10 s.
18 The tubes were decanted again on the bench for approximately 10 min.
19 The excess “dirty” HBSS was transferred to the same falcon as in Step 16.
20 Steps 16, 17, and 18 were repeated once or until a clear supernatant was obtained.
  Pellet washes are important to ensure the removal of exocrine tissue debris in the contents because contamination increases the level of proteases, which influence islet viability in subsequent steps.
  We suggest collecting the lavages in larger falcon tubes to examine the liquid later and ensure that no islets have passed into these supernatants before discarding the contents.

#### 2.5.3 Collection of isolated islets

21 Approximately 3 mL of HBSS solution was added to a black‐bottomed Petri dish. The dark background of the plate makes it easier to see the white islets.
22 The plate was positioned on the binuclear stereomicroscope.
23 The digested contents were poured.
24 The islets were manually removed from the plate using a pipette with a 200‐μL tip.

This manual selection was performed to select, quantify, and allocate islets for subsequent analyses (e.g., in vitro incubation, PCR, WB). The number of islets varied according to the age, size, diet of the animals, and the correct execution of the technique. Ideally, while one person performs the cannulation process, another person should perform the digestion so that the tissue does not remain longer than necessary in each stage, and a third person should collect the islets.

The falcon tubes containing the digested islets may be stored in a Styrofoam container with ice until all of the islets have been collected, but only after the digestion process has been completed (Step 20).

Because this technique is a delicate and time‐consuming process, we recommend the use of a maximum of 10 animals per day. For in vitro incubations, 6 animals per day may be used.

Once successfully isolated, islets may be used in various experimental and clinical processes, such as beta cell purification, gene expression analysis, islet beta cell maturation, proliferation, cell stress responses, cell survival, metabolism, and functional maintenance of GSIS.

## 3 RESULTS

### 3.1 Insulin secretion from isolated pancreatic islets

After isolation of pancreatic islets, several techniques may be performed (WB and RT‐PCR) as previously mentioned. The isolation of pancreatic islets from adult mice has been used for new techniques recently, such as the generation and long‐term expansion of functional islet organoids in vitro (Wang et al., 2022). However, the technique of incubating islets with different stimuli or in vitro stimulation will provide many answers about the functioning and physiology of these cells, primarily for investigating β‐cell dysfunction.

**FIGURE 7:**
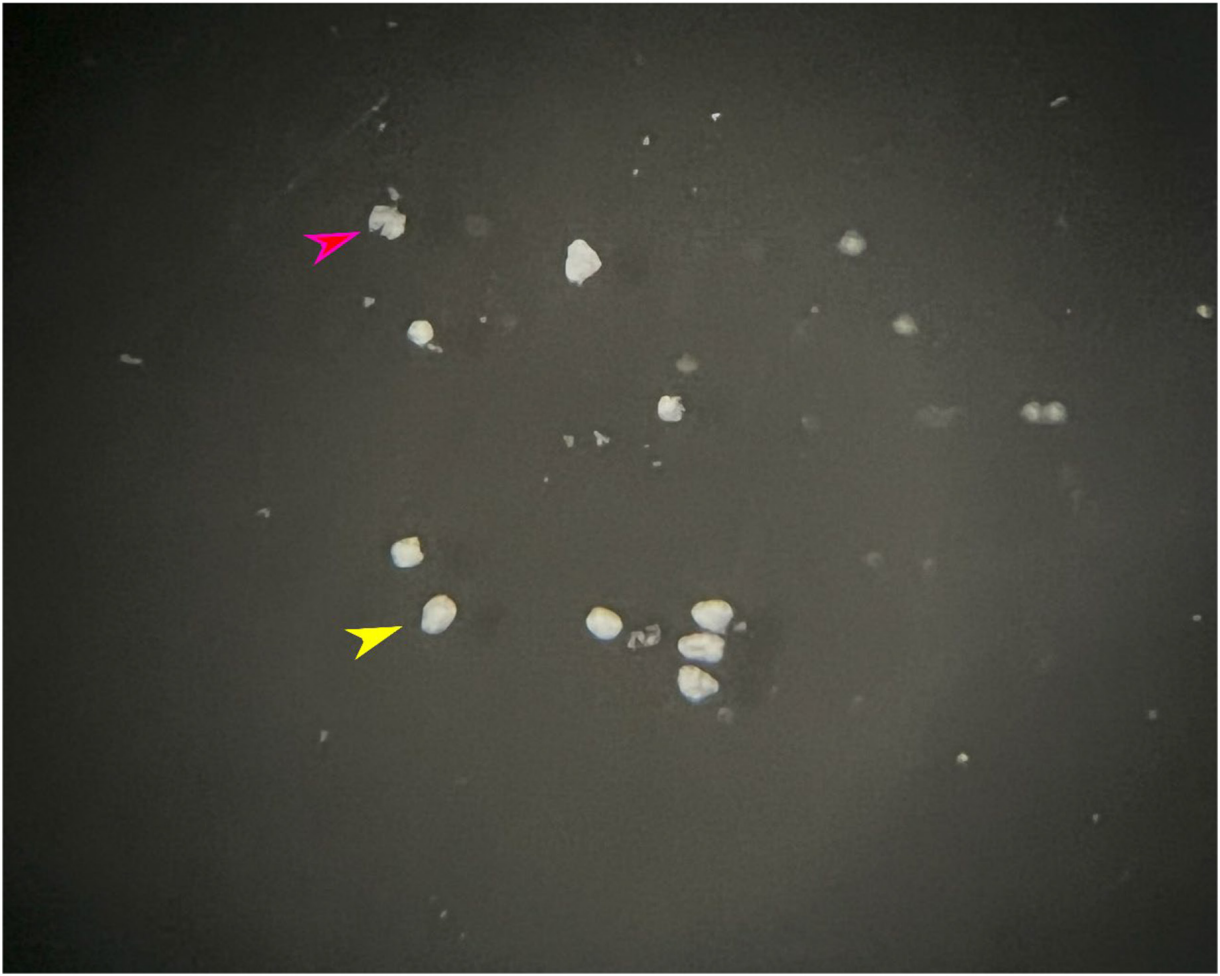
Image of the petre plate with a black background containing the HBBS solution and the pancreatic islets. Signed by the yellow arrow we can see a perfect islet, with its shape and structure preserved. Already marked by the pink arrow, a very digested islet is represented, misshapen with its structure broken.

After collection of the isolated islets, they may be separated into groups of approximately 4 islets, such as the ones indicated by the yellow arrow in Figure 7 (the amount must be standardized for all groups in the experiment), in 24‐well plates containing different solutions to stimulate insulin secretion. The first step for activation of insulin secretion after the entire digestion protocol is a pre‐stimulatory stabilization period of this group of islets with a basal glucose solution of 5.6 mM diluted in Krebs‐Ringer bicarbonate buffer (Alcazar & Buchwald, 2019; Scott et al., 1981). The time of this preincubation varies from a minimum of 30 to a maximum of 60 min (Miranda et al., 2017; Pietrobon et al., 2020), and the quantity is approximately 1 mL/well. Preincubation and incubation must be performed under controlled temperature conditions (37°C) and agitation, and, optionally, under oxygenation, to maintain cell viability.

We describe one example of an islet incubation protocol using different glucose concentrations. However, an infinite number of possibilities for stimulating pancreatic islets is possible. It is important to consider that when stimulating the islet to analyze insulin secretion, the solution will always be based on a glucose concentration, and this concentration must be compatible with in vivo stimulation. For example, for islet stimulation with an adrenergic agonist, a base buffer with a high glucose concentration of 16.7 mM is needed (de Oliveira et al., 2016). For some secretagogues of interest, in addition to requiring a specific glucose concentration for enhanced insulin secretion, it is necessary to add a reagent to inhibit degradation enzymes, such as with stimulation with acetylcholine muscarinic receptor agonists or antagonists. Acetylcholine is constantly degraded by acetylcholinesterase. Therefore, it is necessary to add an inhibitor of this enzyme, such as neostigmine, to the buffer (de Oliveira et al., 2018; Grassiolli et al., 2007).

#### 3.1.1 Krebs‐Ringer bicarbonate buffer (KRBB)

It is necessary to make three solutions, and each one must be added to 1 L of distilled water:

1. NaCl (sodium chloride)—26.890 g
2. NaHCO_3_ (sodium bicarbonate)—8.065 g
  - KCl (Potassium chloride)—0.490 g.
  - MgCl (magnesium chloride)—0.813 g.
3. CaCl_2_ (calcium chloride)—0.588 g

To prepare the solution, 250 mL of solutions 1, 2, and 3 plus 250 mL of distilled water were added. One gram of BSA was added to 1 L of solution, and the pH was adjusted to 7.4. The pH must be adjusted with HCl because it is generally approximately 9. Once made, this solution can only be stored for 1 week under refrigeration at 4°C because the pH is quite variable.

KRBB is used as a base to incubate islets with different glucose concentrations, which range between low and supraphysiological (e.g., 5.6–27 mM) or for other secretagogues. For incubations with subsequent glucose concentrations, the amount of glucose to be added to the KRBB must be calculated considering the amount of buffer needed and the concentration of interest.

After removal of the volume of preincubation liquid, the volume of incubation solution containing the stimulus of interest (approximately 1 mL) was immediately added. The plate was incubated for a minimum of 30 and a maximum of 60 min under controlled conditions, as mentioned above. The liquid was removed using a 1000‐μL pipette, and the wells were always observed under a stereomicroscope to ensure that no islets were removed. The liquid was stored in a −20°C freezer for later insulin dosing.

Insulin measurement can be performed using radioimmunoassay (RIA) or specific ELISA kits for mice or rats, following the manufacturer's instructions (Carlsson et al., 2010).

Isolated islets can also be used for culture over longer periods, therefore, to ensure that there is no microbial contamination, it is necessary to add antibiotics to the culture medium. As already described, the most used are gentamicin, penicillin, streptomycin, tetracycline, neomycin, erythromycin, and chloramphenicol (Shewade et al., 2001). When used in the correct proportion (generally 1% is added to the medium) (Oh et al., 2022; Saliba et al., 2017), antibiotics or antimycotics do not affect the viability and function of pancreatic islets.

### 3.2 Troubleshooting

1. *Buffer preparation:* The main error during this step is failure to measure the pH or maintaining an incorrect pH of the solutions. This step is critical for the viability and maintenance of intact pancreatic islet structures. Take care when handling the solutions and always keep the solutions on ice. Failure to add BSA and HEPES to the collagenase solution also reduces the success of islet isolation.
2. *Pancreatic perfusion:* Frequent errors include failure to perfuse the pancreas completely and long perfusion times, which cause tissue degradation. To ensure complete perfusion of the pancreas, the final portion of the pancreatic duct (portion next to the duodenum) must be clamped. However, avoiding clamping the portion of the duct before the junction with the duodenum because closing it too much also disrupts perfusion and prevents the collagenase solution from reaching the caudal portion of the pancreas. This mistake causes the loss of a large part of the total number of islets.
3. *Pancreas digestion:* The main mistakes at this stage are inattention to the specific digestion time of your sample, the use of incompatible collagenase types with pancreatic tissue, and failure to maintain the perfused tissue under agitation and at a controlled temperature (37°C). After digestion, do not vigorously shake the tube containing the tissue (avoid using magnetic stirrers). Understanding how your sample reacts to collagenase digestion is important. Therefore, perform tests before starting the experiments. Digestion times that are too long or too short will harm your experiment. The effectiveness of the technique depends on controlled digestion patterns.
4. *Islet in vitro incubation:* Failure to respect the incubation times for secretion, failure to preincubate the isolated islets in a basal glucose solution, and storing the supernatant containing islets (after stimulation) are the most frequent errors. Be careful and respect the time needed for each process.

## 4 DISCUSSION

This protocol arose from carrying out the existing protocols and improving the cannulation technique, solutions (e.g., a large amount of albumin in the solution), times, and steps until obtaining a 100% effective method for mice and rats, with their individualities. Our methodology is simple in terms of solutions and steps compared to the existing ones. It does not rely on specific equipment and all solutions are manually made, which can be interesting from a low‐cost point of view.

Our protocol showed more advantages than others described once the 30G needle (13 × 0.3 mm) connected to the syringe for mice enables this technique for rats of different ages, including those that are small, approximately 21 days old (weaning age), using a cannulation technique like that of adult mice by size compatibility between the two species at these different ages, with enough viable isolated islets for molecular analyses. Moreover, most of the methodologies described remove the animal's pancreas and chop it with scissors or perform cannulation through the ampulla of Vater, which is unfeasible for mice and small rats due to the impossibility of identifying the ampulla. As for adult rats, our experience with cannulation through the ampulla of Vater results in fewer islets than the present protocol.

Another difference in the present protocol is that because islets are not distributed evenly throughout the pancreas, taking advantage of the animal's anatomy is essential to extract islets from the entire organ. It is important to highlight that the tail region of the pancreas, close to the spleen, can be well reached in both mice and rats by perfusion through the duct (as we performed). This correctly inflated region provides an excellent result regarding the number of isolated islets, provided that the remaining steps were followed correctly, such as the digestion step. Moreover, our protocol does not require a reagent in the decantation process, which could affect islet viability.

Herein, we used only male animals to demonstrate the technique. However, there is no methodological modification to apply this technique of pancreas cannulation and islet isolation in female animals. What can vary between the sexes and must be observed by the researcher is that females generally are a little smaller than males, which can impact the amount of collagenase and HHBS used during the procedure and the pancreas incubation time after collagenase perfusion, as a smaller pancreas requires less digestion time. Consideration must be given to the influence of the gonadal axis on insulin secretion and glycaemic homeostasis in females. Therefore, it is necessary to evaluate the phase of the estrous cycle in which this animal is on the day of the experiment. It is more common to choose a phase in which gonadotropins and sex hormones are not elevated, such as dioestrus (Andersson et al., 2013).

## 5 CONCLUSION

Throughout this protocol, we provided step‐by‐step images to improve understanding. This isolation method achieved our objective of obtaining a good number of islets with excellent viability for culture or molecular biology techniques. However, it is important to make some adjustments according to each situation to create ideal conditions for isolation. Notably, the isolation time, the number of islets, and the success of the technique, are influenced by numerous factors, as demonstrated throughout the text. If unexpected results are encountered, adjust the digestion time or other factors. Adjust one step at a time to identify which variable is the problem.

## FUNDING INFORMATION

This work was supported by Fundação Carlos Chagas Filho de Amparo à Pesquisa do Estado do Rio de Janeiro, FAPERJ (grant numbers: E‐26/200.914/2022 for P.C.L., and E‐26/200.984/2022 for V.S‐M.), and Conselho Nacional de Desenvolvimento Científico e Tecnológico, CNPq (grant numbers: 309507/2021‐9 for P.C.L. and 303785/2020‐9 for V.S‐M.).

## CONFLICT OF INTEREST STATEMENT

The authors have no conflicts of interest to disclose.

## ETHICS STATEMENT

The method described here was approved by the Animal Experimentation Ethics Committee of the State University of Rio de Janeiro (CEUA, UERJ 020/2022).

## Data Availability

Data sharing is not applicable—no new data are generated.
